# Recent advances in the surgical care of breast cancer patients

**DOI:** 10.1186/1477-7819-8-5

**Published:** 2010-01-20

**Authors:** Alessandra Mascaro, Massimo Farina, Raffaella Gigli, Carlo E Vitelli, Lucio Fortunato

**Affiliations:** 1 Department of Surgery, Senology Unit, San Giovanni-Addolorata Hospital, Via Amba Aradam, 9, 00187 Rome, Italy

## Abstract

A tremendous improvement in every aspect of breast cancer management has occurred in the last two decades. Surgeons, once solely interested in the extipartion of the primary tumor, are now faced with the need to incorporate a great deal of information, and to manage increasingly complex tasks.

As a comprehensive assessment of all aspects of breast cancer care is beyond the scope of the present paper, the current review will point out some of these innovations, evidence some controversies, and stress the need for the surgeon to specialize in the various aspects of treatment and to be integrated into the multisciplinary breast unit team.

## Introduction

No other solid cancer has witnessed such a tremendous change and improvement in terms of diagnosis and management as breast cancer in the last 2 decades. This remains the most common cancer among women worldwide [1].

Breast cancer management has become increasingly complex, and requires a comprehensive assessment of multiple tasks in addition to the simple extirpation of the primary tumor, including breast imaging, advanced pathology, nuclear medicine and a variety of adjuvant therapies, both local and systemic. This has shifted breast cancer treatment into a multidisciplinary science.

Only a few decades ago, women with breast cancer were uniformly treated with radical mastectomy and total axillary dissection to achieve good loco-regional control and the possibility of full recovery. Conservative and selective surgical approaches to the breast and to the axilla, once viewed with scepticism, have now become standard of therapy for most patients [2,3].

Earlier detection and more effective treatments have resulted both in an increasing percentage of small breast cancers found at the initial diagnosis and in a small decline in mortality [2].

Howewer, as the current goal for breast cancer patients is "conservation" instead "the more radical excision the better", the impact of local recurrence on survival remains a relevant issue, and is presently a subject of research and debate.

The aims of this review are to analyze the most important changes which have occurred in the last several years in the surgical management of breast cancer patients and to review some relevant issues such as sentinel lymph node biopsy, the impact of local therapy on survival, and the aesthetic results.

### Non Palpable Lesions and Localization Techniques

Breast cancer screening has dramatically increased the diagnosis of suspicious, non-palpable breast lesions, and therefore also the need to localize them in order to plan surgical treatment [4]. Furthermore, patients with a breast cancer removed with clear margins at the first excision seem to have a decreased risk of local recurrence compared with patients who need further re-excisions to achieve negative margins [5].

This represents a "hot" topic in breast surgery, since approximately 50% of breast cancers in modern surgical practices are non palpable, and this incidence is certainly destined to increase [6].

Today, pre-operative confirmation of malignancy is almost always achieved by fine-needle or core-biopsy, and therefore, we need to localize these small cancers to allow a one-step precise and directed excision.

Compared with their palpable counterpart, non-palpable lesions are associated with both a lower stage of disease and a substantially decreased incidence of lymph node involvement [7].

Wire localization (WL) is the most common technique used to identify small nodules, microcalcifications or parenchymal distorsions. Howewer, it has some disadvantages such as pain and discomfort in some patients, and occasionally carries risks of complications including dislodgement of the wire, intraoperative wire transection, retention of wire fragments, thermal injury with the use of cautery, hematoma and even syncope. WL is performed in most institutions as an additional procedure, outside the operating room, with further problems related to organization and scheduling.

Successful localization with free margins of resection is not always achieved with this technique and failures, with consequent re-excisions, are reported in up to 33% of cases [6-10].

A precise localization of a breast tumor with the wire is not always possible, and the angle of access and trajectory depends, in part, on the radiologist's ability. Furthermore, the introduction of the wire directly above the lesion may be technically problematic, especially under stereotaxic guidance in locations such as the inferior quadrants.

For this reasons several new techniques have been introduced in order to achieve breast tumor localization.

Radioguided occult lesion localization (ROLL) is a useful method to detect nonpalpable lesions through the injection of a nuclear tracer (99 m TC-labelled colloidal albumin) directly around the tumor under ultrasound or stereotaxic guidance. Then, the excision of the primary tumor is guided by a gamma probe, and a sentinel node biopsy can be performed at the same time if needed [11-13].

Unlike the WL, the procedure is generally more straightforward and well tolerated, and the success rate is reported to be very high [14-23] (Table 1).

**Table 1:** Complete excision rate of non palpable lesions by ROLL

Author	Year	N	Complete excision rate (%)
Gennari [14]	2000	647	99

Tanis [15]	2001	45	87

Ronka [16]	2004	215	93

Thind [17]	2005	68	84

Van Rijk [18]	2007	293	89

Moreno [19]	2008	61	93

Medina-Franco [20]	2008	50	89

Lavouè [21]	2008	72	85

Van Esser [22]	2008	40	78

Sarlos [23]	2009	100	98

Although ROLL has been shown to be comparable to WL in at least 2 restrospective [24,25] and four prospective-randomized studies [19,20,22,26] with regards to the ability to identify the lesion, four reports have demonstrated a statistical difference in achieving tumor-free margins in favor of the former technique [17,24,26,27].

Another technique for localization of non-palpable breast tumors is represented by intraoperative ultrasound (IOUS).

It satisfies most requirements for an ideal technique to localize non-palpable breast tumors which are well visualized by ultrasound, while directing planes of surgery during the excision. This in turn is helpful in guaranteeing both negative margins and an adequate contour of resection in order to minimize the volume of excision.

Identification rate of non-palpable lesions and free margins of resection obtained through this procedure are extremely high [28-34] (Table 2).

**Table 2:** Identification rate of small lesions by US

Author	Year	N	Identification (%)	Free Margins (%)
Harlow [28]	1999	65	100	97

Smith [29]	2000	81	100	96

Kaufman [30]	2003	100	100	90

Bennett [31]	2005	103	100	93

Ngo [32]	2007	70	96	94

Haid [33]	2007	299	100	100

Fortunato [34]	2008	77	100	97

Furthermore, microcalcifications, usually visible only by mammography, are sometimes associated with sonographic alterations that can be detected, and removal of such lesions under ultrasound guidance can sometimes be performed [35].

### Implications of Local Therapy

As conservative approaches have developed in the last three decades and represent the standard of care for breast cancer patients around the world, the incidence of local recurrence (LR) has been widely studied. It occurs in 5-10% of patients at 10 years, and it is more pronounced in the first 3 or 4 years after primary surgery [36,37].

Although several factors have been associated with the risk of LR, at the multivariate analysis only age, status of surgical margins and postoperative radiotherapy seem to be independently correlated with it [38]. Patients with multifocal tumors, once uniformely thought to be associated with a higher risk of LR, and therefore treated with mastectomy, are now often offered breast conservation, when technically feasible, as most studies seem to indicate that the LR rate is not higher in these cases than previous reports for unifocal cancers [39]. Similarly, infiltrating lobular carcinoma is probably not associated with a higher incidence of LR compared to the ductal counterpart if resected with negative margins [40].

The influence of age on the risk of LR is striking, and many reports have shown that this is increased three-fold for women less than 40 years of age [38,41-43]. Furthermore, younger patients show a statistically significant reduction of LR in several "boost trials", again demonstrating the importance of an appropriate local therapy particularly in this age group [44].

It is interesting to note that despite the widespread use of conservative approaches in breast cancer patients, there is no general agreement even on the definition of "negative" margins, and many describe such as the absence of tumor at the microscopic or inked margin, or with 1-3 mm clearance. It is clear that a high percentage of patients whose tumors are 2-5 mm from the radial margins have residual disease at re-excision [45]. For this reason, and despite best efforts, as many as 20-25% of patients in many institutions around the world return to the operating room after initial surgery for re-excision [46]. While many reports fail to describe a statistically significant impact of margins on LR, most would agree that one of the primary goals of conservative surgery is the removal of the primary tumor with a portion of normal breast tissue, so as to maintain a good breast shape [47-54].

Although the results of six prospective randomized trials in patients with invasive breast cancer have demonstrated that lumpectomy/quadrantectomy plus RT and mastectomy have equivalent survival results [55-60], it is worthwhile to remember that the first conservation trial, the Guy's wide excision study initiated in the 60's, has shown a decreased survival in the group treated conservatively [61]. This suggests that poor surgical removal of the primary tumor, possibly with dubious margins and without inking of the specimen, together with employment of suboptimal post-operative radiotherapy, may lead to a negative impact not only on local control but also on survival [57,61].

Although additional retrospective data has been accumulated in the last few years suggesting that failure of local control has an impact on survival [62], the most striking evidence comes from the EBCTCG meta-analysis [63].

This has shown that adjuvant RT after BCS not only may improve local control, but it may also reduce 15-year breast cancer mortality. The effect of radiation on LC seems more pronounced in node positive patients, while the effect on survival remains important both for node-negative and node-positive patients [64,65].

This has lead many to suggest that for every four women for whom local failure is prevented, one life can be saved. As this disease is prevalent, and LR after quadrantectomy and radiation is far from being an exceptional event, this seems quite an important issue.

Minimalistic approaches are no longer viable for women with breast cancer, and the aim of the contemporary surgeon is to team up with all available specialists, and to coordinate efforts to reach the goal of local control.

### Skin Sparing Mastectomy

Although breast conservation surgery (BCS) has become the gold standard for patients with early breast cancer, mastectomy remains an option and it is necessary in at least 20% of those women with multicentric tumors, widespread DCIS, and large or recurrent tumors [66]. Sometimes the risk of an unpleasant cosmetic result with conservative surgery to achieve tumor-free margins, or personal desire to avoid radiation therapy plays a role in the decision process.

New options are now available for these women and they represent the forefront of the surgical therapy for breast cancer patients.

Oncologic need to remove the skin envelope or the nipple-areola complex has never been proved, and has been lately challenged on solid evidence and background. Immediate breast reconstruction (IBR), a procedure once discouraged for some years after primary surgery because of fear of relapse, is now performed routinely for an increasing number of patients. This has a profoundly positive psychological effect, and allows for a more solid recovery of these women so touched by this disease [67].

Skin sparing mastectomy (SSM) has been increasingly used in the last 15 years to improve cosmesis because the skin envelope is preserved and the surgical access is limited to a small elliptical incision around the areola [68]. Our understanding that skin involvement is rare is corroborated by pathologic studies, and when present, it is usually over the primary tumor site, or is found in cases with advanced disease, skin tethering, or lymphatic emboli [69]. However, as maximal skin preservation is desirable, special technical considerations are to be addressed by the surgeon because the risk of leaving some glandular tissue behind can be as high 10% if skin flaps are more than 5 mm thick [70].

Clinical experience has confirmed so far that SSM has very acceptable results in terms of local control even in those studies with longer follow-up and is comparable to modified radical mastectomy both in terms of local control and survival [71-86] (Table 3).

**Table 3:** Recurrence Rates after SSM

Author	Year	N	LR (%)	F/U (mo)
Slavin [71]	1998	51	2	45

Newman [72]	1998	372	6	25

Simmons [73]	1999	77	4	60

Kroll [74]	1999	114	7	72

Rivadeneira [75]	2000	71	6	49

Medina-Franco [76]	2002	176	4	73

Foster [77]	2002	67	4	49

Carlson [78]	2003	565	5	65

Greenway [79]	2005	225	2	49

Margulies [80]	2005	50	0	8

Yano [81]	2007	124	2	34

Patani [82]	2008	83	0	34

Scholz [83]	2008	72	0	42

Ueda [84]	2008	74	5	50

Garwood [85]	2009	64	1	13

Gerber [86]	2009	238	10	101

Complications after SSM and immediate breast reconstruction are reported in about 15% of cases, and include flap necrosis and implant loss [87-89].

However, this risk must be weighed with the advantage in cosmetic result and in patient satisfaction (as defined by perception of body image, social activity and sexual aspects), because these outcomes are better in SSM with IBR compared with radical mastectomy [84].

We favor IBR in almost all cases, and therefore routinely perform SSM to allow the plastic surgeon to intervene more comfortably at the same time. Sometimes, post-operative radiation therapy may be needed, and although several studies and current clinical recommendation report that the rate of complication is too high if an implant is inserted in this setting [74,90-92], in recent years a few studies have reassessed this issue [93-96]. We believe that this is still an option in selected cases, as it allows the patient to start more readily adjuvant systemic therapies if needed, and when it fails, it does not preclude or negatively influence possible autologus conversion or final outcome.

### Nipple-Sparing Mastectomy

"Nipple sparing mastectomy" (NSM) is the ultimate challenge of this process which aims for an interaction between conservative techniques and radical surgery. In this procedure, the skin flap covering the breast gland and the nipple-areola complex (NAC) are preserved. In some cases the major ducts are removed.

In the past, the nipple has been routinely removed for fear of occult tumor involvement, although this has probably been overestimated. Many clinical studies have shown that this involvement varies from 6 to 23% depending on the size of the primary tumor, its location, multicentricity, lymph node positivity and the presence of extensive intraductal component [97-102] (Table 4).

**Table 4:** Occult Histologic Nipple Involvement

AUTHOR	YEAR	PATIENTS (N)	NIPPLE INVOLVEMENT (%)
Santini [97]	1989	1291	12

Laronga [98]	1999	286	6

Sikand [99]	2005	220	7

Vlajciz [100]	2005	108	23

Petit [101]	2006	106	10

KG [102]	2008	397	15

We believe that this occurrence is rare in modern clinical practice and although the risk is real, patients can probably be safely selected for this approach.

Nevertheless, exact indications and contraindications to this procedure are not well defined, and the incidence of nipple involvement is reported to be as high as 50% for tumors measuring more than 4 cm or located closer than 2 cm from the nipple [103]. Therefore, the best candidates for NSM are patients with no large tumor (T1-T2), with lesions at least 1 cm from the areola or 2 cm from the nipple, or small multicentric carcinomas [101].

Furthermore, nipple involvement is rare if the retroareolar margin is free of disease [104].

A strategic issue is to avoid partial or total nipple or areola necrosis because, although this can be easily treated postoperatively and under local anesthesia, it results in psychological distress to the patient, and it must be considered a failure of the procedure itself.

The rate of nipple necrosis varies from 0 to 15% [101,105-110] (Table 5).

**Table 5:** Nipple Necrosis after NSM

AUTHOR	YEAR	N	PARTIAL NECROSIS %	TOTAL NECROSIS %
Crowe [105]	2004	48	6	0

Caruso [106]	2006	50	2	0

Sacchini [107]	2006	192	7	4

Petit [101]	2006	106	10	5

Komorowski [108]	2006	38	5	8

Stolier [109]	2008	82	0	0

Voltura [110]	2008	51	6	0

Surgical technique is extremely important. It is now well understood that the use of periareolar incisions should be abandoned, as it negatively affects the vascular supply of the nipple-areola complex, and that either a radial or a lateral incision seem to be more effective in this regard [109].

Although it is not clear how much tissue can or should be left under the NAC, or if "nipple coring" (removal of the terminal ducts from the inside of the nipple papilla) should be performed (and how aggressively), results of NSM can been examined in a few retrospective studies published so far, and the local recurrence rate is shown to be quite low in the majority of them [103,106,107,110-115] (Table 6).

**Table 6:** Nipple Sparing Mastectomy: Local Recurrence

AUTHOR	YEAR	N	LOCAL RECURRENCE %	FOLLOW-UP (months)
Simmons [111]	2004	17	0	24

Caruso [106]	2006	50	2	66

Sacchini [107]	2006	123	2	25

Denewer [112]	2007	41	0	8

Crowe [113]	2008	149	3	41

Voltura [110]	2008	51	6	18

Sookhan [103]	2008	18	0	11

Gerber [114]	2009	61	12	101

Petit [115]	2009	1001	1.4	20

The role of post-operative radiotherapy following NSM is unknown at the present, although a three-fold decrease in the rate of locoregional failure has been reported in one series [116]. However, in this retrospective study only large tumors (> 3 cm) were included, and the site of failure is not clearly described.

Proponents at the European Institute of Oncology have recently updated their experience reporting on 1,001 patients treated with a single intra-operative radiotherapy treatment (21 Gy) with electrons (ELIOT) to the NAC after NSM in the assumption that this single radiation dose may sterilize occult cancer foci eventually left in the glandular tissue behind the areola [115]. This is the largest experience with NSM, to date, and the incidence of local recurrence is reported at 1.4% with a median follow-up of 20 months. Although some concerns have been raised regarding the possible negative effects (even long-term) on the vascularity of the NAC after a single large dose of radiotherapy, the usefulness of this approach is appealing but currently unproven. Of interest, in a subgroup of patients, treated with ELIOT, with very close tumor margins under the areola, no local recurrence was observed.

### Oncoplasty

Oncoplasty has been developed in the last 15 years as a new surgical approach and incorporates a variety of relatively simple, common plastic techniques. This has generated much enthusiasm around the world, among both by breast and plastic surgeons, and in the UK formal oncoplasty training has been developed [117].

Indeed, oncoplastic surgery represents a step forward in breast conservation, allowing us to treat tumors in problematic locations (for example in the lower quadrants), to avoid poor cosmetic results, asymmetry or unpleasant scarring in the upper quadrants, and to obtain wider excisions and tumor free margins [118].

Oncoplasty is safe, as no statistical differences in terms of local relapse and disease-free survival are evidenced when comparing classic quadrantectomies and oncoplastic approaches [118-120]. It should be considered for those patients where adequate local excision cannot be achieved without a significant risk of local deformity, as it frequently occurs in resection of more than 20% of breast volume, or for tumors located in the central, medial or inferior quadrants. Other indications include women considering a breast reduction in addition to excision.

Several volume displacement techniques can be employed, including glandular remodelling, inferior or superior pedicle flaps, round block excision, and the Grisotti flaps. Their description is beyond the scope of this review.

Centrally located tumors account for 5 to 20% of breast cancer cases and have long been thought to be associated with a higher incidence of multicentricity and multifocality [121,122]. However, other more recent reports have failed to substantiate a specific correlation between location of the tumor and multicentricity [123,124]. For this reason, they represent an important challenge for breast surgeons, as they have been classically treated with a mastectomy, and until few years ago only 7% of central breast cancers were treated with conservative surgery [119].

Several studies on the local recurrence rate after central quadrantectomy, each with a small number of patients, show very acceptable results even long-term [125-132] (Table 7).

**Table 7:** Local Recurrence after Central Quadrantectomy

AUTHOR	YEAR	N	LR %	FOLLOW-UP (Months)
Galimberti [125]	1993	37	0	32

Haffty[126]	1995	98	6	108

Simmons[127]	2001	32	6	60

Pezzi[128]	2004	15	6	32

Tausch [129]	2005	44	7	51

Naguib [130]	2006	23	9	13

Huemer[131]	2007	31	0	34

Wagner [132]	2007	31	0	42

A direct comparison between central quadrantectomy and mastectomy has seldom been studied, and no significant differences in terms of local failure and overall survival have been reported [119,127,133-135]. However, these reports are limited by their retrospective nature and may not be comparable because mastectomy was usually performed for larger tumors. Only one prospective non-randomized study has been published so far, and it has confirmed an equivalent outcome in terms of local or systemic disease [132].

We believe that by adhering to the principles of breast-conserving surgery, including complete resection of the primary tumor with a negative margin, these centrally located tumors can be treated adequately by nipple-areolar resection. Adjuvant radiation therapy to the remainder of the breast can treat subclinical microscopic disease, if present, with accepTable local control and adequate cosmesis.

### Sentinel Lymph node Biopsy, and Management of Special Circumstances

Lymph node involvement is the single most important prognostic factor for survival in breast cancer patients, and consequently information about it provide both staging information and guidance regarding treatment options [136].

SLN biopsy is now considered an adequate axillary staging procedure for patients who have breast cancer because it is easy and reproducible if carried out by experienced clinicians, and carries less morbidity compared to axillary node dissection [137].

Many concerns were raised in the past because SLN biopsy can result in some false-negative cases. A recent meta-analysis of 69 trials found the rate of false negatives to be about 7% of the node-positive patients [138].

Much of what is known today regarding SLN biopsy in breast cancer does not result from randomized trials. The procedure has been accepted quickly by most dedicated surgeons around the world on the basis of a growing body of evidence that SLN is effective. Often, patient demand has overcome the caution that surgeons usually demonstrate before abandoning a well-tested procedure, such as axillary node dissection. In some cases, randomized trials have been prematurely closed because of problems in accrual, either because randomization was not acceptable to patients, or because surgeons, after acquiring sufficient experience with SLN biopsy, were unwilling to allow their patients to enter the trial.

Enhanced pathology of the SLN has generated much confusion and even controversy, but it is a key point as different results can be obtained by different groups using different protocols. A survey of the European Working Group for Breast Screening Pathology reported that 240 pathologists replying to a questionnaire described some 123 different pathology protocols [139].

**Figure 1:**
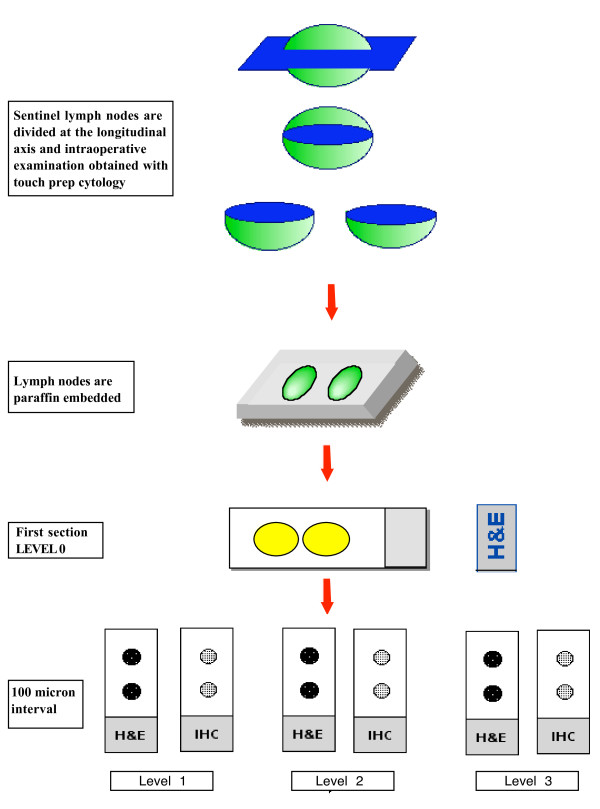
**A simple and standardized protocol, with slicing at three levels at 100-micron intervals and double staining with both hematoxylin-eosin and immunohistochemistry, that has allowed the pathologists in the authors' group to diagnose additional nodal disease with an increment of nearly two thirds compared with standard, single-section analysis of the lymph nodes stained with hematoxylin-eosin**. (Adapted from Fortunato L, Amini M, Costarelli L, et al. A standardized sentinel lymph node enhanced pathology protocol (SEPP) in patients with breast cancer. J Surg Oncol 2007;96[6]:471; with permission.)

The authors' group recently has proposed a simple, practical standardized protocol, with slicing at three levels at 100-micron intervals and double staining with both hematoxylin and eosin (H&E) and immunohistochemistry (MNF116) (Figure 1) [140]. This protocol has allowed our pathologists to increase the diagnosis of additional nodal disease by nearly two-thirds compared with standard, single-section analysis of the lymph nodes stained with H&E, although the majority of this gain is represented by minimal disease, micrometastases or isolated tumor cells. (Figure 1)

Some important issues, such as the prognostic influence of SLN micrometastases, and the use of SLN biopsy in special circumstances are still subject of open debate among clinicians.

The prognostic significance of micrometastases in SLN is controversial. Its diagnosis is rapidly increasing (17% per annum since 1997) as reported by a recent analysis of the SEER database of 175,000 patients treated between 1990 and 2002 [141]. This probably results from a combination of factors, including the diagnosis of smaller tumors by mammographic screening, and the implementation of SLN biopsy with more frequent diagnosis of minimal node involvement by step sectioning.

In the most important retrospective study, conducted by the International (Ludwig) Breast Cancer Study Group, 9% of 921 patients who had negative axillary lymph nodes on routine H&E single-section analysis were found to be node positive on serial sectioning [142]. In some, but not in all, groups these women had a significantly poorer 5-year disease-free and overall survival rate. Recent data seem to confirm the hypothesis that micrometastases are indeed a marker of poorer prognosis.

In a review of the published literature in 1997, Dowlatshahi [143] analyzed all large and long-term studies and confirmed a statistically significant decrease in survival associated with the presence of axillary node micrometastases. The group at Memorial Sloan Kettering Cancer Center has used serial sections and immunohistochemistry to re-evaluate all axillary lymph nodes from 373 patients operated in the 1970s who were deemed to be node negative by routine histopathology [144]. The presence of any detectable micrometastatic disease was associated with decreased disease-free and overall survival rates.

In a review of 1959 cases treated at the European Institute of Oncology from 1997 to 2000, Colleoni and colleagues [145] have found that minimal involvement (micrometastases or isolated tumor cells) of a single lymph node correlated with decreased disease-free survival and doubled the risk of distant metastases.

Recently, the presence of isolated tumor cells or micrometastases in the SLN were found to be associated with a reduced 5-year disease-free survival among 856 women in the Netherlands with favorable early-stage breast cancer who did not receive adjuvant therapy. In this study, an additional cohort of 995 patients who received adjuvant therapy showed an improved disease-free survival at a median follow-up of five years [146].

At the present time, surgical management and systemic options in case of SLN micrometastases are controversial. Most retrospective studies have reported a substantial rate of additional lymph node metastases in patients with SLN micrometastases, with a wide range between reports, making one think that patient selection is a key in determing the choice of candidates for completion lymph node dissection [147-154] (Table 8).

**Table 8:** Additional Positive Non Sentinel Metastases for Micrometastatic SLN

AUTHOR	YEAR	SLN (N)	MICROMETASTASES (%)	POSITIVE NON-SLN (%)
Reynolds [148]	1999	220	27	22

Turner [149]	2000	514	42	22

Nos [150]	2003	800	33	7

Hwang [151]	2003	627	21	57

Fan [152]	2005	390	29	17

Rutledge [153]	2005	358	25	3

Schrenk [154]	2005	966	39	18

Van Rijk[155]	2006	2150	30	19

Ongoing or completed/closed randomized trials such as the ACOSOG Z0010, the National Surgical Adjuvant Breast and Bowel Project B32 and the International Breast Cancer Study Group 23-01, will help to fully understand whether further axillary treatment should be mandatory when the SLN is positive [155-157].

There are still a few clinical settings in which SLN biopsy generates controversy, and we would like to review some of them:

### Ductal Carcinoma In Situ (DCIS)

Management of DCIS is clinically relevant, because its incidence is increasing and represents today approximately 20-25% of newly diagnosed cases of breast cancer [158].

Traditionally, axillary node metastases were identified by conventional histology in fewer than 2% of patients whose surgical specimen was interpreted as containing DCIS only, probably because the presence of invasive cancer can be unrecognized [159].

Studies of patients with "pure" DCIS who have undergone SLN biopsy have confirmed an extremely low rate of axillary node involvement [160,161]. Unfortunately, the diagnosis of "pure" DCIS can be misleading because microinvasion can be missed even with an extensive histologic search and immunostaining, and because a preoperative diagnosis is not always feasible due to sampling error after microbiopsy. A recent meta-analysis, including 22 published reports, has estimated that the incidence of SLN metastases in patients with a pre-operative diagnosis of DCIS is 7.4%, compared with an incidence of 3.7% for patiens with a definitive (post-operative) diagnosis of DCIS [162].

In DCIS with diagnosed microinvasion the incidence of axillary metastases has been reported to range from 3% to 10% in small series [163-173] (Table 9).

**Table 9:** SLN Biopsy in DCIS with Microinvasion

AUTHOR	YEAR	N	SLN POSITIVITY (%)
Zavatosky [164]	1999	14	4

Klauber-De More [165]	2000	31	3

Wassergerg [166]	2002	57	3

Intra [167]	2003	41	10

Le Bouedec [168]	2005	107	7

Sakr [169]	2006	128	7

Zavagno [170]	2007	43	9

Fortunato [171]	2007	77	8

Doyle [172]	2009	145	5

Rubio [173]	2009	47	4

Polom [174]	2009	183	5

In case of SLN involvement after diagnosis of DCIS, it is not clear whether a complete axillary node dissection should be performed, or additional systemic therapy be considered. A review of 21 series collected only 29 such patients undergoing axillary lymphadenectomy after a positive SLN finding, and no additional metastases were found after completion of lymphadenectomy [174].

### Recurrent Breast Cancer

Approximately 10% of breast cancer patients are expected to experience an ipsilateral recurrence 10 to 15 years after their initial treatment.

Although patients who have an ipsilateral recurrence of breast cancer are at increased risk of systemic relapse, their prognosis is not uniformly bad, and approximately two thirds of patients are alive at 5 years [175]. Until recently, axillary re-evaluation was not indicated in these cases.

Recent studies, however, have suggested that a repeat SLN can be performed after a previous SLN biopsy, and sometimes after an axillary node dissection. This has the potential to alter clinical management, as it may help to stratify the risk of systemic disease, and to consider the need of additional systemic therapies.

For a recurrent breast cancer, a repeat SLN biopsy seems more successful after a previous SLN biopsy than after an axillary node dissection, and in this setting SLN positivity is not uncommon [176-185] (Table 10).

**Table 10:** SLN in Recurrent Breast Cancer

Author	Year	N	Success after previous SLND (%)	Success after previous ALND (%)	Extra-axillary localization of SLN (%)	Positive SLN (%)
Sood [177]	2004	4	-	4/4	2/4	0/4

Agarwa l [178]	2005	2	-	2/2	2/2	1/2

Roumen [179]	2006	12	2/2	8/10	7/12	4/10

Newman [180]	2006	8	1/8	7/7	10/10	0/7

Taback [181]	2006	15	5/6	6/9	8/15	3/11

Intra [182]	2007	65	65/65	-	5/63	7/63

Port [183]	2007	46	-	22/46	13/46	10/64

Barone [184]	2007	19	6/7	0/12	16/19	2/16

Axelsson [185]	2007	46	-	22/46	13/46	7/22

Koizumi [186]	2008	31	3/31	16/31	14/23	4/28

**TOTAL**		**248**	**82/119 (69)**	**87/167 (52)**	**90/240 (37)**	**38/227 (17)**

The risk of an extra-axillary localization (parasternal, interpectoral, or supraclavicular region or to the contralateral axilla) is reported in approximately one-third of cases, particularly after a previous AND.

### Neoadjuvant Chemotherapy

An area of particular interest is the use of SLN biopsy in patients undergoing neoadjuvant chemotherapy, because the number of patients choosing this option is increasing.

Until recently, feasibility and accuracy of SLN biopsy in these patients were considered limited due to the possible alteration of lymphatic patterns after chemotherapy, but several studies have reached different conclusions.

Data reported in the literature show an identification rate from 71 to 100% and a false-negative rate less than 13% [186-205] (Table 11).

**Table 11:** Sentinel Lymph node biopsy after neoadjuvant chemotherapy

Author	Year	N	Identification rate (%)	False-negative rate (%)
Breslin [187]	2000	51	84	13

Tafra [188]	2001	29	93	0

Fernandez [189]	2001	40	85	22

Julian [190]	2002	34	91	0

Stearns [191]	2002	34	85	23

Brady [192]	2002	14	93	0

Schwartz [193]	2003	21	100	9

Piato [194]	2003	42	98	12

Reitsamer [195]	2003	30	87	7

Kang [196]	2004	54	72	11

Lang [197]	2004	53	94	4

Shimazu [198]	2004	47	94	12

Balch [199]	2004	32	97	5

Mamounas [200]	2005	428	85	12

Tausch [201]	2006	167	85	8

Newman [202]	2007	54	98	8

Shen [203]	2007	69	93	25

Kinoshita [204]	2007	104	93	10

Hino [205]	2008	55	71	0

Classe [206]	2009	195	90	11

**TOTAL**		**1553**	**1345/1553 (87%)**	**68/538 (13%)**

Our group, however, favors SLN biopsy before beginning of neoadjuvant therapy, as pathologic stage, along with complete response, are still the most important prognostic factors for these patients who so frequently belong to a young age group. Securing stage allows a more precise knowledge of the risk for the single patient; it allows meaningful comparison between different neoadjuvant protocols; and in case of negativity, it allows a simple tumorectomy after therapy for those patients with good responses.

### Multicentric Breast Cancers

Multicentric breast cancer may occur in up to 10% of cases. SLN biopsy is also accurate in these patients, because SLN drains the whole breast, regardless of tumor localization, as reported by many studies [206-216] (Table 12).

**Table 12:** SLN biopsy in multicentric breast cancers

Author	Year	N	Identification rate (%)	False negative rate (%)
Schrenk [207]	2001	19	100	0

Fernandez [208]	2002	53	98	0

Kumar [209]	2003	59	93	0

Tousimis [210]	2003	70	96	8

Goyal [211]	2004	75	95	9

Knauer [212]	2006	150	91	4

Ferrari [213]	2006	31	100	8

Gentilini [214]	2006	42	100	NR

D'Eredita [215]	2007	30	100	6

Cipolla [216]	2008	34	100	0

Lo Yf [217]	2009	135	100	0

**TOTAL**		**698**	**666/698 (95%)**	**11/190 (6%)**

In the largest report to date, a study from the Austrian Sentinel Node Study Group, a retrospective comparison between 142 patients with multicentric and 3,216 patients with unicentric cancers, showed no difference in detection of the SLN, or false-negative rates [211]. Therefore, we believe that SLN should be considered standard of care for these tumors.

Although either multiple Tc-99 injections or a single intradermal injection over the largest-size lesion has been described, a single periareolar injection of the tracer has been proposed as a mean to simplify this technical aspect, and there evidence that this leads to the identification of a single, representative SLN [212].

### Internal Mammary Sln Biopsy

Although prospective randomized trials have not demonstrated a therapeutic benefit of removal of internal mammary lymph nodes (IMN) in patients with breast cancer [217], it is well known that involvement of this chain is associated with worse prognosis. Furthermore, medial and inferior tumors have been reported to drain more commonly to IMN [218], although this has not been routinely taken in consideration in the last decades. Indeed, the IMN represents an important pathway, draining lymphatics from the deep breast lobules along the pectoral fascia and intercostals muscles [219].

Several studied have shown that SLN biopsy of the IMN is feasible, although it requires mapping through a deep intraparenchimal or peritumoral injection, as IMN identification is almost impossible after an intradermal injection [220,221]. The procedure involves more commonly a direct exposure of the second or third intercostal space, division of the intercostal muscle fibers, and is associated with the rare possibility of breach of the pleural cavity [222]. This has raised concerns regarding the acceptability of this procedure if there is no definitive demonstration of a survival benefit.

Studies have evidenced that SLN of IMN can be identified in 8-34% of breast cancer patients, and it can potentially benefit 7-15% of such patients because of a positive histologic finding [220,222-226]. Therefore, a potential change in management in the whole group is uncommon.

In case of IMN positivity adjuvant radiotherapy or systemic therapy may be offered, and clinical trials would be needed to determine whether it improves survival.

### Breast Units: A Challenge For The Clinician

In the past, a few studies [227-234] have analyzed various high-risk surgical procedures (such as pancreatic or hepatic surgery) and correlated post-operative outcomes to hospital or surgeon procedure volume. The results of these studies have strongly suggested that complex visceral resections ought to be regionalized and concentrated in high volume hospitals.

Surprisingly, this rule may also apply to breast cancer care, because even if the surgical skills required in most cases are not usually complex, the need for a comprehensive, multidisciplinary management does seem to play a difference. This has prompted a debate regarding how to guarantee women with the best care possible through a preferential access to specialized breast cancer centers.

An analysis of some 233,000 operated breast cancer patients extracted from a nationwide US database and operated over a 13-year period has shown that the risk of death was three times higher for patients treated at low-volume hospitals, and that they were less likely to receive breast conservation. Furthermore, the risk of post-operative complications was higher and length of stay was longer in this group [235].

A review of 24,834 patients from the Florida Cancer Data System reported higher survival rates for patients treated at teaching hospitals compared with community or low-volume hospitals [235,236]. It was concluded that much of these differences were due to the decreased use of proven adjuvant therapies, again underlining the need for an integrated treatment for this disease.

Not only hospital volume and type, but also surgeons' experience, do make a difference. In a report of almost 30,000 patients operated in the Los Angeles County, treatment by a surgical oncologist (a "specialist") resulted in a 33% reduction in the risk of death at 5 years at the multivariate analysis [237].

In the US this information has resulted in a rapid increment of Breast Fellowship, recognizing that appropriate training is one of the key factors in improving quality of care. Currently, the number of such subspecialties almost equals that for surgical oncology. Nevertheless, until few years ago 25% of surgeons in the US performed almost 90% of the surgery for breast cancer, and probably this occurs even more frequently around the world [238].

In Europe, the Florence and Hamburg [239,240] statements have anticipated these findings as early as 1988, and, through a joint effort of EORTC, the European Society of Mastology (EUSOMA) and Europa Donna, the innovative concept for standard guidelines of Breast Units has been proposed to assure the best quality of care to women with breast cancer.

The EUSOMA "Requirements of a Specialist Breast Unit" was first published in 2000 and sets mandatory criteria for accreditation. This revolutionary concept is based on a process of voluntary accreditation; it was established because hospitals will likely be eager to claim that they have specialized breast units, and specialists will wish to show that they work in recognized units.

Requirements for accreditation indicate the need of one Breast Unit every 250,000 total population, and include at least 150 new cases of breast cases diagnosed each year, a core team in which each member must have special training in breast cancer (surgeon, radiologist, oncologist, pathologist, patient support staff, data managers, psychologist, genetist), regular multidisciplinary case management meetings, and adequate treatment facilities for patients.

We now know that a service provided by a trained specialist is more efficient and more cost effective; diagnostic decisions are made earlier and unnecessary investigations avoided; operations conducted by specialists produce better results for technical reasons; the interpretation of imaging techniques and the reading of histology is much more likely to produce definitive opinions if carried out by experts.

All this is leading towards a radically different type of organization for the treatment of breast cancer. This change will be driven not much from "mandatory" requirements, but by the willingness of more sophisticated breast cancer patients to search for the most appropriate treatment and the best possible results.

## Conclusions

The "new era" of breast cancer treatment began more three decades ago with the revolutionary concept of breast conservation, and has not yet finished.

Clinical research, multidisciplinary approaches, and sophisticated therapies are being sought by every women newly diagnosed with breast cancer and hopefully will be more accessible so we can improve the overall quality of care for breast cancer treatment.

Surgeons must keep up with this process, and lead future changes to reach the goal of complete recovery for every patient.

## Competing interests

The authors declare that they have no competing interests.

## Authors' contributions

All Authors participated in the design and coordination of the study, read and approved the final manuscript.
